# Association of HSD11B1 polymorphic variants and adipose tissue gene expression with metabolic syndrome, obesity and type 2 diabetes mellitus: a systematic review

**DOI:** 10.1186/s13098-015-0036-1

**Published:** 2015-04-28

**Authors:** Filipe Valvassori do Nascimento, Vanessa Piccoli, Mayara Abichequer Beer, Anize Delfino von Frankenberg, Daisy Crispim, Fernando Gerchman

**Affiliations:** Division of Endocrinology, Hospital de Clínicas de Porto Alegre, Rua Ramiro Barcelos 2350, Prédio 12, 4° andar, Bairro Santana, Porto Alegre, RS 90035-003 Brazil; Postgraduate Program in Medical Sciences: Endocrinology, Faculdade de Medicina, Universidade Federal do Rio Grande do Sul, Rua Ramiro Barcelos 2400, 2° andar, PPG Endocrinologia, Bairro Santana, Porto Alegre, RS 90035-003 Brazil

**Keywords:** Diabetes mellitus, Obesity, Metabolic syndrome, *HSD11B1*, Polymorphisms, Gene expression

## Abstract

The *HSD11B1* gene is highly expressed in abdominal adipose tissue, and the enzyme it encodes catalyzes the interconversion of inactive cortisone to hormonally active cortisol. Genetic abnormalities of *HSD11B1* have been associated with the development of abnormal glucose metabolism and body fat distribution. To systematically review studies evaluating the association of *HSD11B1* gene expression in abdominal adipose tissue and *HSD11B1* polymorphisms with obesity, the metabolic syndrome (MetS), and type 2 diabetes (T2DM), we conducted a search in MEDLINE, SCOPUS, and Cochrane Library databases in April 2015. The inclusion criteria were observational studies (cross-sectional, cohort, or case–control), conducted in adults, which analyzed the relationship of *HSD11B1* polymorphisms and/or *HSD11B1* expression in abdominal adipose tissue with obesity, MetS, or T2DM. Of 802 studies retrieved, 32 met the inclusion criteria (23 gene expression and 9 polymorphism studies). Twenty one studies analyzed the relationship between abdominal subcutaneous and/or visceral *HSD11B1* expression with central and/or generalized obesity. Most studies reported that abdominal adipose *HSD11B1* expression increased with increasing body mass index (15 studies) and abnormalities of glucose metabolism (7 studies), and varied with the presence of MetS (3 studies). Nine studies analyzed the association of 26 different *HSD11B1* polymorphic variants with obesity, MetS, and T2DM. Only an Indian study found an association between a polymorphic variant at the *HSD11B1* gene with MetS whereas in Pima Indians another polymorphic variant was found to be associated with T2DM. While the literature suggests that *HSD11B1* is hyperexpressed in abdominal adipose tissue in subjects with obesity and abnormal glucose metabolism, this seems to be not true for *HSD11B1* gene expression and MetS. Although an association of polymorphic variants of *HSD11B1* with MetS in Indians and in the T2DM population of Pima Indians were found, most studies did not find a relationship between genetic polymorphic variants of *HSD11B1* and obesity, MetS, and T2DM. Their reported conflicting and inconclusive results, suggesting that polymorphic variants of HSD11B1 may have only a small role in the development of metabolic abnormalities of susceptible populations in the development of MetS and T2DM.

## Introduction

Hydroxysteroid (11-beta) dehydrogenase type 1 (11-βHSD1) is a bidirectional enzyme, encoded by the *HSD11B1* gene, that is highly expressed in liver and adipose tissue. It normally converts the inactive hormone cortisone into its active form cortisol, acting as a reductase [1].

Overexpression of the *HSD11B1* gene in adipocytes has been shown to be related to high cortisol concentrations in adipose tissue and to the development of central obesity, insulin resistance (IR), and diabetes in mouse models [2]. On the other hand, *HSD11B1* knockout mice exposed to a high fat diet are protected against the development of obesity and hyperglycemia [3]. Moreover, 11-βHSD1 inhibitors have been shown to be effective in treating different aspects of the metabolic syndrome (MetS), promoting weight loss and reducing IR and hyperglycemia.

Cushing’s syndrome, which is caused by excess glucocorticoid production, has clinical features that resemble those of MetS in several aspects, suggesting that they share possible pathogenic pathways which result in their metabolic abnormalities. As overexpression of *HSD11B1* in abdominal adipose tissue is associated with increased adipose tissue cortisol concentrations, polymorphic variants of this gene may be related to MetS development [4].

To elucidate this issue, we conducted a systematic review of the literature addressing the potential association of *HSD11B1* polymorphic variants and abdominal *HSD11B1* adipose tissue expression with MetS, type 2 diabetes mellitus (T2DM), and obesity.

### Methods

The Preferred Reporting Items for Systematic Reviews and Meta-Analysis (PRISMA) statement was used in this report. This systematic review is registered in PROSPERO with number CRD42014008705 and can be assessed on http://www.crd.york.ac.uk/PROSPERO/display_record.asp?ID=CRD42014008705#.VQmwC9LF9A0.

#### Search strategy, study selection, and data extraction

Observational studies (cross-sectional, cohort, or case–control) which have analyzed the relationship of polymorphisms and/or *HSD11B1* gene expression with obesity, MetS, or T2DM in human adults were considered eligible. Exclusion criteria were: subjects under 18 years, the presence of malignancy, infectious diseases or diseases that may affect *HSD11B1* gene expression or was described to be associated with its polymorphic variants such as Cushing’s syndrome, osteoporosis, cortisone reductase deficiency (CRD), Alzheimer’s disease and polycystic ovary syndrome (PCOS). A literature search was performed in MEDLINE, Cochrane Central, and Scopus databases. We also manually searched the references of published studies. Study selection was not limited by language. The search strategy was based on the following Medical Subject Headings (MeSH): ((((“Obesity“[Mesh]) OR ”Diabetes Mellitus, Type 2”[Mesh]) OR “Prediabetic State”[Mesh])) AND (“11-beta-Hydroxysteroid Dehydrogenase Type 1”[Mesh]).

Study selection was performed by two independent investigators (FVN and VP). Disagreements were resolved by discussion and consensus; when necessary, a third reviewer (FG) was consulted. Data was extracted by two investigators (FVN and VP) using a standardized form. The information extracted from each individual study was as follows: design, ethnicity, polymorphisms genotyped, tissue site for biopsy and gene expression analysis, number of individuals in each group, gender distribution, body mass index (BMI), waist circumference, waist-to-hip ratio, and fasting and 2-h plasma glucose levels after a 75-g oral glucose tolerance test. The Newcastle–Ottawa Scale (NOS) was used to assess the quality of the selected studies [5]. The NOS contains eight items categorized into three dimensions, including selection, comparability, and exposure. A series of response options is provided for each item. A star scoring system is used to allow semi quantitative assessment of study quality, such that the highest-quality studies are awarded a maximum of one star for each item, except on the comparability item, for which two stars can be assigned. The total NOS score thus ranges from zero to nine stars.

## Results

**Figure 1:**
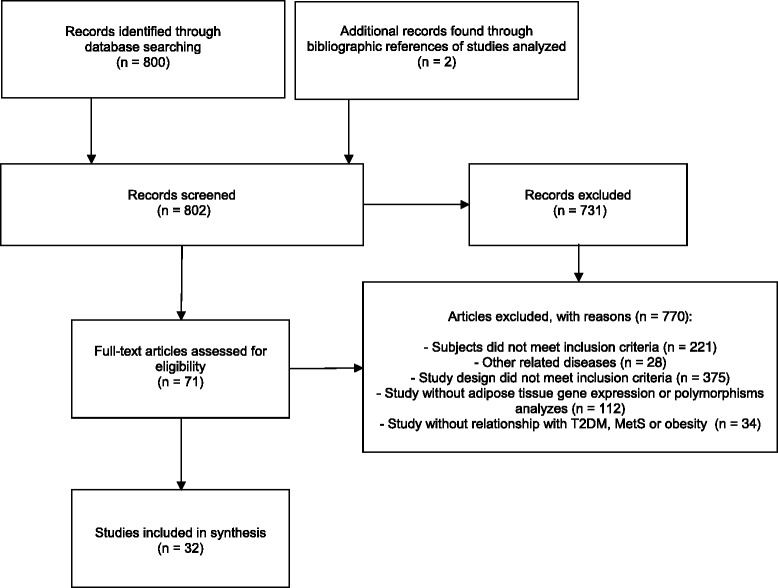
Study selection flow diagram.

Our search strategy yielded 802 records (Figure 1). On the basis of titles and abstracts, we selected 71 studies for full-text examination, of which 32 fulfilled the final inclusion criteria. Studies were grouped according to the relationship they set out to assess: 1) *HSD11B1* gene expression in subcutaneous/visceral abdominal fat and MetS [6-8]; 2) *HSD11B1* gene expression in subcutaneous/visceral abdominal fat and hyperglycemia [6,8-14]; 3) *HSD11B1* gene expression in subcutaneous/visceral abdominal fat and obesity [4,6-11,13,15-28]; 4) *HSD11B1* polymorphisms and obesity, MetS, or T2DM [29-37]. The main results of these studies are presented in Tables 1, 2, 3 and 4. The mean quality score was 5.31 stars, and the details of quality assessment were described in Table 5.

**Table 1:** **Relationship between** ***HSD11B1*** **gene expression in abdominal adipose tissue and the metabolic syndrome: studies and subjects’ characteristics**

**Author, year**	**Subjects**’ **characteristics**	**T2DM**	**Obesity**	**BMI (kg/m** ^**2**^ **)**	**MetS**	**Expression in VAT (AU)**	**Expression in SAT (AU)**
Alberti, 2007 [6]	n: 62	12 (19.4)	62 (100.0)	37.4 ± 5.1	25 (40.3)	MetS+: 2.6 ± 0.5	MetS+: 2.0 ± 1.5
	Female: 52 (83.9)						
	Age: 44.4 ± 11.1					MetS-: 1.7 ± 1.0	MetS-: 0.7 ± 0.4^a^
	Ethnicity: N/D						
Muñoz, 2009 [8]	n: 32	6 (18.8)	32 (100.0)	36.7 ± 3.8	16 (50.0)	MetS+: 7.5	MetS+: 11.6
	Female: 21 (65.6)					MetS-: 10.3	MetS-: 12.3
	Age: 40.2 ± 12.3						
	Ethnicity: N/D						
Michalaki, 2012 [7]	n = 27	2 (7.4)	19 (70.4)	46.1 ± 6.6	11 (40.7)	Lean controls: 27.8 ± 16.0	MetS+: 86.5 ± 29.8
						MetS+: 62.9 ± 24.4	
						MetS-: 107.2 ± 77.7^b^	MetS-: 155.9 ± 124.9
	Female: 27 (100)						
	Age: 37.3 ± 9.7						
	Ethnicity: N/D						

Data expressed as absolute and relative frequencies or mean ± standard deviation as appropriate. T2DM: type 2 diabetes mellitus; BMI: body mass index; MetS: metabolic syndrome; VAT: visceral adipose tissue; SAT: subcutaneous adipose tissue; AU: arbitrary units; N/D: not described. ^a^P < 0.01 vs. MetS+ group. ^b^P < 0.01 vs. lean controls.

**Table 2:** **Relationship between** ***HSD11B1*** **gene expression in abdominal adipose tissue and hyperglycemia: studies and subjects’ characteristics**

**Author, year**	**Subjects’ characteristics**	**T2DM**	**Obesity**	**BMI (kg/m** ^**2**^ **)**	**MetS**	**Expression in VAT**	**Expression in SAT**
Lindsay, 2003 [13]	n: 31	N/D	N/D	35.4 ± 7.4	N/D	N/D	r = 0.46, P < 0.05 with HOMA-IR
	Female: 14 (45.2)						
	Age: 28.8 ± 7.7						
	Ethnicity: 12 Caucasian						
	19 Pima Indian						
Goedecke, 2006 [11]	n: 26	0 (0)	N/D	27.2 ± 0.9	N/D	r = −0.13, P = NS with fasting glucose	r = 0.40, P < 0.016 with fasting glucose
	Female: 26 (100)						
	Age: 41 ± 2.0						
	Ethnicity: 11 Caucasian						
	8 Mixed race						
	7 Black						
Alberti, 2007 [8]	n: 62	12 (19.4)	62 (100.0)	37.4 ± 5.1	25 (40.3)	N/D	r = 0.460, P < 0.0001 with fasting glucose
	Female: 52 (83.9)						
	Age: 44.4 ± 11.1						
	Ethnicity: N/D						
Tomlinson, 2008 [14]	n = 101	7 (6.9)	101 (100)	34.4 ± 4.3	N/D	N/D	r = 0.44, P < 0.005 with glucose AUC
	Female: 66 (65.3)						
	Age: 48.0 ± 7.0						
	Ethnicity: N/D						
Muñoz, 2009 [8]	n: 32	6 (18.8)	32 (100.0)	36.7 ± 3.8	16 (50.0)	r = −0.19, P = NS with fasting glucose	r = 0.15, P = NS with fasting glucose
	Female: 21 (65.6)						
	Age: 40.2 ± 12.3						
	Ethnicity: N/D						
Baudrand, 2010 [9]	n = 49	11 (22.4)	49 (100)	42 ± 6.1	31 (63.3)	r = 0.48, P = 0.005 with fasting insulin	N/D
	Female: 35 (71)						
	Age: 42.2 ± 10.1						
	Ethnicity: N/D						
Baudrand, 2011 [11]	n = 41	6 (14.6)	41 (100)	36.5 (36.5 – 47.0)	N/D	r = 0.37, P = 0.025 with fasting glucose	N/D
	Female: 29 (70)						
	Age: 41.0 (34.0 – 47.5)						
	Ethnicity: N/D						
Gyllenhammer, 2014 [12]	n = 36	N/D	36 (100)	35.2 ± 0.6	N/D	N/D	r = −0.574, P = 0.140 with disposition index
	Female: 23 (63.9)						
	Age: 21.5 ± 0.4						
	Ethnicity: 16 African-Americans						
	20 Hispanics						

Data expressed as absolute and relative frequencies or mean ± standard deviation or median (p25 – p75) as appropriate. T2DM: type 2 diabetes mellitus; BMI: body mass index; MetS: metabolic syndrome; VAT: visceral adipose tissue; SAT: subcutaneous adipose tissue; N/D: not described; AUC: area under the curve in oral glucose tolerance test; NS: non-significant.

**Table 3:** **Relationship between** ***HSD11B1*** **gene expression in abdominal adipose tissue and obesity: studies and subjects’ characteristics**

**Author, year**	**Subjects’ characteristics**	**T2DM**	**Obesity**	**BMI (kg/m** ^**2**^ **)**	**MetS**	**Expression in VAT**	**Expression in SAT**
Paulmyer-Lacroix, 2002 [21]	n: 30	N/D	18 (60)	33.8 ± 5.3	N/D	N/D	BMI < 25 kg/m^2^: 1.6 (HA)
	Female: 27 (90)						BMI ≥ 30 kg/m^2^: 4.0 (HA)^a^
	Age: 37.8 ± 11.2						
	Ethnicity: N/D						
Tomlinson, 2002 [26]	n: 32	0 (0)	11 (34.4)	28.1 ± 0.7	N/D	BMI < 30 kg/m^2^: 15.5 ± 0.3 (n-fold)	BMI < 30 kg/m^2^: 15.5 ± 0.4 (n-fold)
	Female: 32 (100)						
	Age: 43 (28 – 65)					BMI ≥ 30 kg/m^2^: 14.8 ± 0.5 (n-fold)	BMI ≥ 30 kg/m^2^: 15.1 ± 0.5 (n-fold)
	Ethnicity: N/D						
Wake, 2003 [27]	n: 32	0 (0)	N/D	25.9 ± 0.9	N/D	N/D	r = 0.63 with BMI^c^
	Female: 16 (50.0)						
	Age: 55.5 ± 3.0						
	Ethnicity: Caucasian						
Lindsay, 2003 [13]	n: 31	N/D	N/D	35.4 ± 7.4	N/D	N/D	r = 0.34 with BMI^a^
	Female: 14 (45.2)						
	Age: 28.8 ± 7.7						
	Ethnicity: 12 Caucasian						
	19 Pima Indian						
Engeli, 2004 [16]	n: 70	0 (0)	14 (20)	29.0 ± 3.1	N/D	N/D	BMI < 25 kg/m^2^: 0.5 (AU)
	Female: 70 (100)						BMI ≥ 25 kg/m^2^: 0.9 (AU)^a^
	Age: 57 ± 4						
	Ethnicity: Caucasian						
Goedecke, 2006 [11]	n: 26	0 (0)	N/D	27.2 ± 0.9	N/D	r = 0.35 with VAT volume	r = 0.57 with VAT volume^a^
	Female: 26 (100)						
	Age: 41 ± 2.0						
	Ethnicity: 11 Caucasian						
	8 Mixed race						
	7 Black						
Desbriere, 2006 [15]	n: 22	0 (0)	12 (54.5)	30.2 ± 3.8	N/D	BMI < 25 kg/m^2^: 0.5 (AU)	BMI < 25 kg/m^2^: 0.5 (AU)
	Female: 22 (100)					BMI ≥ 30 kg/m^2^: 2.2 (AU)^a^	BMI ≥ 30 kg/m^2^: 3.6 (AU)^a^
	Age: 36.2 ± 7.5						
	Ethnicity: N/D						
Mariniello, 2006 [19]	n: 24	N/D	8 (33.3)	34 ± 3.4	N/D	BMI < 30 kg/m^2^: 1 (n-fold)	N/D
	Female: 17 (70.8)					BMI ≥ 30 kg/m^2^: 13 (n-fold)^b^	
	Age: 42 ± 10						
	Ethnicity: N/D						
Alberti, 2007 [6]	n: 62	12 (19.4)	62 (100.0)	37.4 ± 5.1	25 (40.3)	BMI ≥ 30 kg/m^2^: 2.1 ± 0.9 (AU)	BMI ≥ 30 kg/m^2^: 1.4 ± 0.8 (AU)
	Female: 52 (83.9)						
	Age: 44.4 ± 11.1						
	Ethnicity: N/D						
Makkonen, 2007 [18]	n: 20	0 (0)	10 (50)	28.7 ± 1.4	N/D	N/D	BMI < 25 kg/m^2^: 0.3 ± 0.1 (n-fold)
	Female: 20 (100)						
							BMI ≥ 25 kg/m^2^: 0.6 ± 0.1 (n-fold)^a^
	Age: 36.5 ± 3.5						
	Ethnicity: Caucasian						
Michailidou, 2007 [20]	n: 21	N/D	N/D	32.7 ± 1.5	N/D	r = 0.57 with BMI^c^	r = 0.58 with BMI^a^
	Female: 21 (100)						
	Age: 35 ± 1						
	Ethnicity: Caucasian						
Paulsen, 2007 [22]	n: 40	0 (0)	20 (50)	34.7 ± 3.8	0 (0)	BMI < 30 kg/m^2^: 0.01 (n-fold)	BMI < 30 kg/m^2^: 0.01 (n-fold)
	Female: 20 (50)					BMI ≥ 30 kg/m^2^: 0.04 (n-fold)^a^	BMI ≥ 30 kg/m^2^: 0.05 (n-fold)^a^
	Age: 41.3 ± 9.8						
	Ethnicity: N/D						
Baudrand, 2010 [9]	n = 49	11 (22.4)	49 (100)	42 ± 6.1	31 (63.3)	N/D	N/D
	Female: 35 (71)						
	Age: 42.2 ± 10.1						
	Ethnicity: N/D						
Muñoz, 2009 [8]	n: 32	6 (18.8)	32 (100.0)	36.7 ± 3.8	16 (50.0)	BMI ≥ 30 kg/m^2^: 7.8 (4.7 – 11.8) (AU)	BMI ≥ 30 kg/m^2^: 11.4 (6.2 – 19.8) (AU)
	Female: 21 (65.6)						
	Age: 40.2 ± 12.3						
	Ethnicity: N/D						
Simonyte, 2009 [24]	n: 27	3 (9.7)	27 (100)	44.6 ± 4.5	N/D	BMI ≥ 30 kg/m^2^: 11.2 ± 4.9 (AU)	BMI ≥ 30 kg/m^2^: 14.1 ± 6.4 (AU)
	Female: 27 (100)						
	Age: 41 ± 8.5						
	Ethnicity: Caucasian						
Zha, 2009 [28]	n: 35	0 (0)	15 (50)	25.5 ± 1.5	N/D	BMI < 25 kg/m^2^: 1.0 (AU)	BMI < 25 kg/m^2^: 1.2 (AU)
	Female: 17 (49)					BMI ≥ 25 kg/m^2^: 1.5 (AU)^c^	BMI ≥ 25 kg/m^2^: 1.5 (AU)^c^
	Age: 49.5 ± 12.5						
	Ethnicity: Chinese						
Svendsen, 2009 [25]	n: 24	N/D	16 (66.7)	30.0 ± 2.7	N/D	N/D	BMI < 25 kg/m^2^: 0.09 (0.03–0.60) (AU)
	Female: 24 (100)						BMI ≥ 25 kg/m^2^: 0.25 (0.09–0.84) (AU)^a^
	Age: 30.7 ± 4.7						
	Ethnicity: N/D						
Simonyte, 2010 [23]	n: 17	N/D	17 (100)	44.4 ± 4.4	N/D	N/D	r = 0.53 with WC^a^
	Female: 17 (100)						
	Age: N/D						
	Ethnicity: N/D						
Baudrand, 2011 [10]	n = 41	6 (14.6)	41 (100)	36.5 (36.5 – 47.0)	N/D	N/D	N/D
	Female: 29 (70)						
	Age: 41.0 (34.0 – 47.5)						
	Ethnicity: N/D						
Michalaki, 2012 [7]	n = 27	2 (7.4)	19 (70.4)	46.1 ± 6.6	11 (40.7)	BMI < 25 kg/m^2^: 27.8 ± 16 (AU)	BMI < 25 kg/m^2^: N/D
						BMI ≥ 30 kg/m^2^: 81.6. ± 46.8 (AU)^a^	BMI ≥ 30 kg/m^2^: 115.7. ± 69.8 (AU)
	Female: 27 (100)						
	Age: 37.3 ± 9.7						
	Ethnicity: N/D						
Leyvraz, 2012 [17]	n = 35	N/D	30 (85.7)	41.3 ± 4.6	N/D	BMI < 30 kg/m^2^: 1.0 (0.6 – 1.4) (AU)	BMI < 30 kg/m^2^: 1.0 (0.8 – 1.2) (AU)
	Female: 35 (100)					BMI ≥ 30 kg/m^2^: 2.9. (2.6 – 3.2) (AU)^c^	BMI ≥ 30 kg/m^2^: 2.2 (2.1 – 2.3) (AU)^c^
	Age: 39.0 ± 9.0						
	Ethnicity: Caucasian						

Data expressed as absolute and relative frequencies, median (interquartile range), or mean ± standard deviation as appropriate. T2DM: type 2 diabetes mellitus; BMI: body mass index; MetS: metabolic syndrome; VAT: visceral adipose tissue; SAT: subcutaneous adipose tissue; HA: hybridized area/field; AU: arbitrary units; WC: waist circumference. ^a^P < 0.05. ^b^P < 0.001. ^c^P < 0.01. r = Pearson’s correlation coefficient. N/D: not described; NS: non-significant.

**Table 4:** **Relationship of** ***HSD11B1*** **gene polymorphic variants with obesity, the metabolic syndrome, and diabetes: studies and subjects’ characteristics**

**Author, year**	**Subjects’ characteristics**	**T2DM**	**BMI (kg/m** ^**2**^ **)**	**MetS**	***HSD11B1*** **polymorphisms**	**Association with metabolic parameters**
Franks, 2004 [30]	n: 918	512 (55.8)	32.7 (28 – 38)	N/D	rs846910 and rs12086634	No association with obesity and diabetes
	Age: 36 (28 – 46)					
	Ethnicity: American Indian					
Nair, 2004 [35]	n: 706 (SNP1), 839 (SNP5)	SNP1: 429 (60.8)	N/D	N/D	SNP1 (rs846910 and rs3334312)	SNP1 – OR = 1.64; P = 0.01
		SNP5: 510 (60.8)			SNP5 (N/D, rs12086634 and rs6752)	SNP5 – OR = 1.34; P = 0.02
						For T2DM (+) vs. T2DM (−)
	Age: N/D					
	Ethnicity: Pima Indian					
Robitaille, 2004 [36]	n: 217	N/D	29.6 ± 4.3	N/D	4478 T > G, 4437–4438insA and 10733G > C	No association with BMI, WC, HDL-c, TG, FPG, fasting insulin, BP, HOMA-IR
	Age: 42.8 ± 7.9					
	Ethnicity: French-Canadian					
Miyamoto, 2009 [33]	n: 3005	N/D	22.8 ± 2.7	431 (14.3)	rs860185, −658G > A, +1930insA, rs12086634, rs2236905, rs2298930, rs932335 and rs6752	No association with MetS
	Age:65.8 ± 10.3					
	Ethnicity: Japanese					
Ku, 2009 [32]	n: 1401	757 (54.0)	24.1 ± 3.0	N/D	rs45441700, rs846908, rs701950, +1932Ginsdel, xrs932335, rs13306422, rs6752 and +30249 T > C	No association with WHR, BMI, FPG, HbA1c, fasting insulin, and HOMA-IR
	Age: 61.8 ± 7.2					
	Ethnicity: Korean					
Moon, 2011 [34]	n: 785	427 (54.4)	23.3 ± 0.2	N/D	rs12086634 and rs1000283	No association with T2DM and MetS
	Age: 62.2 ± 0.5					
	Ethnicity: Korean					
Dujic, 2012 [29]	n: 86	N/D	28.9 (25.7 – 31.5)	43 (50.0)	rs846910 and rs45487298	rs846910 related with ↓ HOMA-IR; P = 0.011^a^
	Age: 47 (40 – 53)					rs45487298 related with ↓ HOMA-IR; P = 0.04^b^
	Ethnicity: Bosnian					
Gandhi, 2013 [31]	n: 205	62 (30.2)	25.7 ± 2.9	105 (51.2)	rs12086634	Associated with MetS (OR = 6.64; P < 0.0001)
	Age: 43 ± 11.5					
	Ethnicity: South Indian					
Turek, 2014 [37]	n: 215	N/D	26.5 ± 4.5	N/D	rs846910 and rs12086634	rs846910 not related with BMI and FPG
	Age: N/D (22 – 72)					rs12086634 not related with FPG; P = 0.06
	Ethnicity: Euro-Brazilian					

Data expressed as absolute and relative frequencies, median (interquartile range), or mean ± standard deviation as appropriate. N/D: not described; T2DM: type 2 diabetes mellitus; BMI: body mass index; MetS: metabolic syndrome; WC: waist circumference; HDL-c: HDL cholesterol; TG: triglycerides; FPG: fasting plasma glucose; BP: blood pressure; HOMA-IR: homeostatic model assessment of insulin resistance; HbA1c: glycated hemoglobin A1c; NPG: non-polymorphic group; PG: polymorphic group. ^a^in MetS (+) group. ^b^in control group.

**Table 5:** **Quality scores of studies included in the systematic review**

**Author, year**	**Case definition**	**Representativeness of the cases**	**Selection of controls**	**Definition of controls**	**Comparability**	**Ascertainment of exposure**	**Total**
Alberti, 2007 [6]	+		+	+	+	++	++++++
Baudrand, 2010 [9]	+	+		+		++	+++++
Baudrand, 2011 [10]	+	+				+	+++
Desbriere, 2006 [15]	+			+		++	++++
Dujic, 2012	+	+	+	+		+++	+++++++
Engeli, 2004 [16]	+	+	+	+		+++	+++++++
Franks, 2004 [30]	+	+	+		+	+++	+++++++
Gandhi, 2013 [31]	+	+	+	+		++	++++++
Goedecke, 2006 [11]	+	+			+	+	++++
Gyllenhammer, 2014 [12]	+	+			++	+	+++++
Ku, 2009 [32]	+	+	+	+	+	+++	++++++++
Leyvraz, 2012 [17]	+	+				+	+++
Lindsay, 2003 [13]	+	+			+	++	+++++
Makkonen, 2007 [18]	+		+	+	+	++	++++++
Mariniello, 2006 [19]	+		+	+		++	+++++
Michailidou, 2007 [20]	+				+	++	++++
Michalaki, 2012 [7]	+			+		++	++++
Miyamoto, 2009 [2009]	+	+	+		+	++	++++++
Moon, 2011 [34]	+	+	+	+	+	+++	++++++++
Munoz, 2009 [8]	+	+				++	++++
Nair, 2004 [35]	+	+	+		+	+++	+++++++
Paulmyer-Lacroix, 2002 [21]	+	+	+	+		+++	+++++++
Paulsen, 2007 [22]	+			+		++	++++
Robitaille, 2004 [36]	+					+++	++++
Simonyte, 2009 [24]	+				+	++	++++
Simonyte, 2010 [23]	+	+			+	+	++++
Svendsen, 2009 [25]	+	+	+	+	++	++	++++++++
Tomlinson, 2002 [26]	+			+		++	++++
Tomlinson, 2008 [14]	+	+			++	+	+++++
Turek, 2014 [37]	+	+			+	++	+++++
Wake, 2003 [27]	+	+			++	+	+++++
Zha, 2009 [28]	+	+		+	+	++	++++++

### Relationship between *HSD11B1* expression in subcutaneous and visceral abdominal fat and the metabolic syndrome

Three studies compared the expression of *HSD11B1* in adipose tissue according to the presence of MetS [6-8] (Table 1). These studies included a total of 121 participants (mean age, 41.7 years). Two of them recruited only obese subjects, whereas the third compared obese vs. lean control subjects; two studies recruited subjects with a predominant female population, whereas one included only women. *HSD11B1* expression was quantified as arbitrary units using similar methodology (RT-qPCR), differing primarily in their housekeeping genes (GUSB, TBP and 18S). Prevalence of T2DM was low and varied between 7.4 and 19.4%. In the first study [6], *HSD11B1* expression in abdominal subcutaneous adipose tissue (SAT) was higher in 62 obese subjects with MetS compared to those without MetS, whereas expression in abdominal visceral adipose tissue (VAT) was similar between groups. Contrasting with these findings, another study with 32 obese individuals (six with T2DM) [8] found that *HSD11B1* expression was not significantly higher in either abdominal SAT or VAT in subjects without MetS compared to those with MetS. On the other hand, in a case–control study, *HSD11B1* expression in abdominal VAT was significantly higher in 19 obese women (two with T2DM) than in eight lean women [7] (Table 1).

### Relationship between *HSD11B1* expression in subcutaneous and visceral abdominal fat and hyperglycemia

Eight studies analyzed the relationship between *HSD11B1* expression in abdominal SAT and VAT with estimates of insulin resistance, β-cell function, blood glucose levels and abnormalities of glucose metabolism and T2DM, in populations of different ethnic background, including Caucasians, African Americans, Pima Indians, Hispanic Americans and populations from Sweden and Denmark (data not clear about ethnicity) (Table 2). One study included only women [11] and seven studies included both genders, mostly women in 6 and mostly men in one [6,8-10,12-14]. The prevalence of T2DM varied between 0 and 22.4% among studies, with two studies where this information was not provided. These differences in gender distribution among studies do not affect the results of these findings. While *HSD11B1* VAT expression increased respectively with increasing fasting plasma glucose [9] and HOMA-IR [10] in morbid obese subjects of two different studies, two have shown similar results with abdominal SAT [11,13]. While comparing the expression of abdominal SAT *HSD11B1* according to glucose tolerance, additional studies have shown that this expression was greater respectively in those with T2DM (1.8 ± 0.7 vs. 1.1 ± 0.4 AU; P < 0.05) [6] and those with impaired glucose tolerance (0.5 ± 0.06 vs. 0.29 ± 0.03 AU; P < 0.005) compared with normal controls [14]. Although in another study, increased abdominal SAT *HSD11B1* expression was related with decreased beta-cell function (r = −0.574) estimated by the disposition index while adjusting for different confounders, this relationship became not significant after the inclusion of hepatic fat fraction in the multivariate model [12]. Finally, one study showed a trend toward increased expression of *HSD11B1* in abdominal SAT in subjects with T2DM compared to normal controls, although the statistical significance of this comparison was not shown due to the small number of subjects with T2DM (n = 6) [8].

### Relationship between *HSD11B1* expression in subcutaneous and visceral abdominal fat and obesity

Twenty one studies assessed the potential relationship of *HSD11B1* expression in subcutaneous and visceral abdominal fat with obesity (Table 3). Six studies did not find a relationship between *HSD11B1* expression in abdominal SAT or VAT and central or generalized obesity [6,7,9,10,24,26]. Conversely, in two studies [15,17], abdominal SAT and VAT *HSD11B1* expression was higher in obese women than in lean women. Similar results were reported in other three studies assessing *HSD11B1* expression only in abdominal SAT of female subjects [16,18,25]. In the first of these studies [15], a greater gene expression in abdominal SAT was also associated with a greater waist circumference and percentage of body fat. In two other studies, *HSD11B1* gene expression was higher in the abdominal VAT of obese subjects than in lean subjects [19,20]. In four studies [8,11,13,27] *HSD11B1* expression in abdominal SAT was positively related with measures of body size and central obesity, namely respectively, BMI [8,13,27] and intra-abdominal VAT volume estimated by MRI [11]. In other two studies [21,22], *HSD11B1* expression in abdominal SAT was higher in obese men and women than in non-obese subjects. In another study, the expression of *HSD11B1* in abdominal VAT was higher in obese women than in lean controls, but this relationship was not assessed in men due to small sample size. Zha et al. found abdominal VAT and SAT *HSD11B1* expression higher in obese subjects than in lean controls as well as a positive correlation between these expressions with BMI [28]. Finally, in a small study sample of women (n = 17), it was found a positive correlation between abdominal SAT *HSD11B1* expression and waist circumference (r = 0.53; P < 0.05), but not with BMI [23].

### Relationship of *HSD11B1* genetic polymorphisms with obesity, the metabolic syndrome, and type 2 diabetes mellitus

Nine studies analyzed the association between 26 different *HSD11B1* polymorphic variants and obesity, MetS, and T2DM (Table 4) [29-37].

Asian populations were assessed in two studies from South Korea [32,34], one study from Japan [33], and one from India [31]. The first study analyzed 757 individuals with and 644 without T2DM [32], and did not find associations between any of four *HSD11B1* polymorphisms with T2DM or MetS. In another study, 427 individuals with and 358 without T2DM were analyzed and no relationship was found between two *HSD11B1* polymorphic variants and T2DM or MetS [34]. In an urban cohort of 3,005 Japanese individuals, seven *HSD11B1* polymorphic variants were assessed and no association with MetS was found [33]. Another study, conducted in 217 French-Canadian men, also failed to find any association between *HSD11B1* polymorphisms and MetS [36]. Finally, in a study of 918 Native Americans of the Gila River Indian Community of Arizona, polymorphic variants of this gene were not associated with T2DM or obesity [30].

In contrast with these negative results, a study conducted in India found a significant risk of MetS in subjects with the *HSD11B1* polymorphism rs12086634 [31]. Another study found a significant risk of T2DM, but not of obesity, in Pima Indians with the *HSD11B1* polymorphic variants rs846910 and rs12086634 [35]. Conversely, another study found that polymorphism rs846910 was associated with decreased insulin resistance (decreased HOMA-IR) in subjects with MetS, and that the rs45487298 polymorphism was associated with similar findings in control subjects without MetS [29]. Finally, it was shown no relationship between rs846910 with BMI or fasting plasma glucose levels in 215 Euro-Brazilian descendants. The authors of the same study concluded that rs12086634 was related with fasting plasma glucose levels in women with a borderline p-value (P = 0.06). However, the sample had limited statistical power, there was no statistical correction for multiple comparisons and no clear definition of what was considered a statistical difference in the methodology section [37].

## Discussion

This systematic review included 32 studies which analyzed the potential relationship of abdominal adipose *HSD11B1* gene expression in human and its polymorphic variants with MetS, T2DM, and obesity.

The relationship between *HSD11B1* expression in abdominal adipose tissue and MetS was analyzed in three studies with distinct population profiles. They did not point out to a relationship between abdominal SAT and VAT *HSD11B1* expression and MetS. However, they were not well designed to answer this question since this was not their main objectives. As a result, these studies might not have enough power to detect differences in *HSD11B1* expression in abdominal SAT and VAT comparing groups of obese subjects with and without MetS without having a lean control group for comparison, as what happened in two of these studies. In the third study that we were able to review, lean control subjects had a significantly lower abdominal VAT *HSD11B1* expression than those presenting the MetS, strengthening this direction of thought. It is our opinion that this does not mean that there is no relationship between abdominal adipose tissue *HSD11B1* and MetS. In other studies it has been shown that a possible relationship between *HSD11B1* enzymatic activity and the MetS exists. It also suggests that this enzymatic activity is more linked to MetS than its enzymatic expression [13,38,39]. In another study, increased omental *HSD11B1* activity in women submitted to a gynecological surgery was associated with larger omental adipocytes, increased lipolysis and lipoprotein lipase activity, decreased high-density lipoprotein cholesterol and adiponectin levels, and an increased homeostasis model assessment of insulin resistance index compared to those with decreased omental activity of this enzyme [38]. Additionally, *HSD11B1* activity was more related with fasting glucose, insulin, BMI and waist circumference than *HDS11B1* expression in abdominal SAT in Pima Indians and Caucasians [13]. Moreover, in a study where *HSD11B1* activity was indirectly studied by a ratio of urinary cortisone to cortisol metabolites, it has been shown a failure to down-regulate HSD11B1 activity in subjects with diabetes compared to normal controls which might contribute to the underlying pathogenesis of the MetS. The lower prevalence of T2DM in the studies analyzed by us may have biased our findings and may explain, in part, why we were not able to find this link [39]. Therefore, no relationship between *HSD11B1* gene expression in abdominal adipocytes and MetS can be conclusively established on the basis of the current literature.

A relationship between *HSD11B1* expression in abdominal adipose tissue and IR, pancreatic β-cell function, fasting glucose, and T2DM were analyzed in eight studies of populations with different ethnic backgrounds [6,8-14]. Their results have shown that increasing *HSD11B1* expression in abdominal VAT and SAT is associated with IR in 2 studies, impaired β-cell dysfunction in one study and increased fasting plasma glucose levels in 4 studies (Table 2). Additionally, two studies have shown that *HSD11B1* expression in abdominal SAT is greater in those with impaired glucose tolerance and T2DM compared with normal controls. Although it is not clear to define the specific compartment of abdominal adipose tissue in which *HSD11B1* expression is mostly associated with metabolic abnormalities of glucose metabolism, these studies suggest that the expression of this gene in abdominal SAT and VAT is related with abnormalities of glucose metabolism and may play a role in the development of T2DM.

The relationship between *HSD11B1* expression in abdominal adipose tissue and markers of central and general obesity was analyzed in 21 studies. While six studies did not find any association between *HSD11B1* expression in abdominal adipose tissue and obesity, fifteen studies found that a greater expression of this gene in VAT and/or SAT was associated with increased BMI, waist circumference, and body fat percentage. The summary data have shown that *HSD11B1* expression in abdominal SAT and VAT is related with central obesity and body size. Unfortunately, we were not able to perform a meta-analysis with the summary of these data, since the studies varies in their way to express BMI (absolute number, lean vs overweight vs obesity, lean controls vs excessive weight), abdominal compartment *HSD11B1* expression (SAT, VAT or SAT + VAT) and different methodologies to quantify this expression (hybridized area/field vs relative curve vs ΔΔ_ct_).

Our collected data suggest that *HSD11B1* polymorphic variants are not associated with MetS, obesity, or T2DM in most studies [30,33-37]. However, in one study, two *HSD11B1* polymorphisms (rs846910 and rs12086634) in linkage with three other polymorphisms were found to be associated with decreased insulin sensitivity, increased plasma glucose levels, and T2DM in Pima Indians [35]. Additionally, the rs12086634 polymorphism was found to be associated with MetS in a study conducted in India [31]. The polymorphic variant rs846910 is located in the promoter region near exon 1 of the *HSD11B1* gene. Carriers of this SNP exhibit increased enzyme activity, which is in agreement with these findings. However, rs12086634 is located in intron 3, and carriers of this polymorphic variant exhibit decreased enzyme activity, which cannot be explained by our findings regarding this SNP. Additionally, the polymorphic variant rs846910 and another one, rs45487298 (SNP localized in intron 3 of the *HSD11B1* gene and related to decreased enzyme expression), were associated respectively with increased insulin sensitivity in subjects with and without MetS in another study [29]. Due to the small sample size to take in account their results (n = 86), it is necessary more solid data to take in account these results. Additionally, since polymorphic variants of *HSD11B1* that change its expression and activity are expected to have a major role in modulating insulin sensitivity and adipose tissue proliferation through regulation of intra-adipocyte cortisol production, the presence of these polymorphic variants would be expected to be associated with obesity as well, which was not demonstrated in the present review [35]. None of studies included in this review assessed how these polymorphic variants affect *HSD11B1* functionality. Since *HSD11B1* activity is dependent on the provision of NADPH by the co-localized enzyme hexose-6-phosphate dehydrogenase (H6PD), polymorphic variants affecting the effects of both H6PD and 11-βHSD1 may be necessary to exert an effect on enzymatic activity, resulting in metabolic abnormalities or a healthy metabolic profile.

## Conclusions

In conclusion, our systematic review have shown that expression of the *HSD11B1* gene in intra-abdominal adipose tissue is probably related to abnormalities of glucose metabolism, T2DM and obesity, with consistent findings across different studies. We were not able to find that expression of the *HSD11B1* gene in intra-abdominal adipose tissue is related with MetS. Finally, the relationship between polymorphic variants of *HSD11B1* with MetS in subjects from India and T2DM in Pima Indians, but not in studies performed in several other populations, suggests that *HSD11B1* polymorphisms may have a small role in the development of these metabolic abnormalities only in these susceptible populations.

## Abbreviations

11-βHSD1: Hydroxysteroid (11-beta) dehydrogenase type 1
IR: Insulin resistance
MetS: Metabolic syndrome
T2DM: Type 2 diabetes mellitus
BMI: Body mass index
NOS: New Castle-Ottawa Scale
SAT: Abdominal subcutaneous adipose tissue
VAT: Abdominal visceral adipose tissue
H6PD: Hexose-6-phosphate dehydrogenase
